# Synthesis of 5-Hydroxy-5-vinyl-2-cyclopentenones, a Family of Rare-Type Natural Products Mostly Recovered from Marine Sources

**DOI:** 10.3390/md23120449

**Published:** 2025-11-23

**Authors:** Yoshihide Usami, Natsuki Asada, Chihiro Shizuma, Karin Negoro, Ryosuke Kawai, Sayaka Kaneda, Noboru Hayama

**Affiliations:** Department of Pharmaceutical Organic Chemistry, Faculty of Pharmacy, Osaka Medical and Pharmaceutical University, 4-20-1 Nasahara, Takatsuki 569-1094, Japan

**Keywords:** synthesis, 5-hydroxy-5-vinyl-2-cyclopentenone, natural product, racemic, conjugate addition, Mislow-Evans rearrangement, microwave-aided reaction

## Abstract

The microwave-aided racemic synthesis of six 5-hydroxy-5-vinyl-2-cyclopentenone-type natural products was achieved. A key reaction involving the construction of the α-keto vinyl carbinol function was realized by applying a Mislow–Evans rearrangement of an allylic sulfoxide, which was prepared by conjugate addition of cyclopentane-1,3-dione-derived enolate to alkynyl sulfoxide to afford 5-hydroxy-3-methoxy-5-vinyl-2-cyclopentenone (**1**). From the common intermediate **1**, five other congeneric natural products were synthesized.

## 1. Introduction

The synthesis of natural products remains an effective method for new drug discovery and therefore attracts significant attention among organic chemists [1,2,3]. Furthermore, several types of natural products still remain unexplored. 5-Hydroxy-5-vinyl-2-cyclopentenones are a family of rare natural products recognized as characteristic fungal metabolites of the *Trichoderma* species. However, they have not been studied in detail, particularly with respect to their bioactivities, owing to their instability or volatility [4].

**Figure 1:**
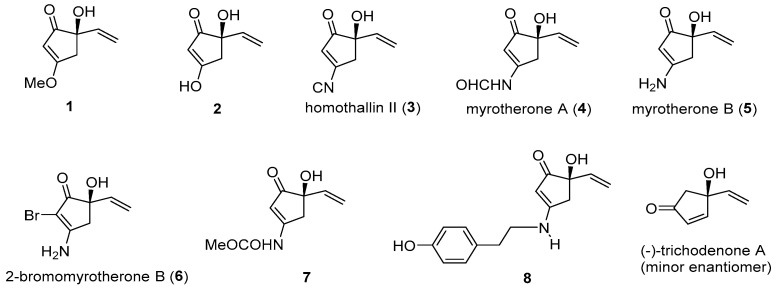
Natural 5-hydroxy-5-vinylcyclopentenones and the related trichodenone A.

The chemical structures of known natural 5-hydroxy-5-vinylcyclopentenones are shown in Figure 1. The first example is 5-hydroxy-3-methoxy-5-vinyl-2-cyclopentenone (**1**), which was discovered from *Trichoderma album* in 1977 [5]. 3,5-Dihydroxy-5-vinyl-2-cyclopentenone (**2**) is an unstable metabolite of *Trichoderma koningii*, which appeared in the literature in 1996 [6]. Homothallin II (**3**) is also a member of this natural product family. The unique chemical structure of **3** of bearing an isocyano group is characteristic. In 1994, Faulls et al. isolated **3** from a UV-irradiated mutant of *Trichoderma haruziunum* and evaluated its antifungal and antibacterial activities [7]. Another study on the isolation of **3** as a metabolite of the *Trichoderma viride* strain H1-7 from marine sediment reported that **3** exhibited significant inhibitory activity against mushroom- and mouse-B16 cell-derived tyrosinase [8]. In 2005, Son et al. isolated myrothenones A (**4**) and B (**5**), along with trichodenone A, as metabolites of the fungal strain *Myrothecium* sp., which was obtained from the marine green alga *Enteromorpha compressa*. Myrothenone A (**4**) exhibited moderate tyrosinase inhibitory activity [9]. Bromomyrothenone B (**6**) was first isolated from a marine algicolous fungus belonging to the genus *Botrytis* [10]. The isolation of **4**–**6** also appeared in another study [11]. Lin et al. reported the isolation of two new 5-hydroxy-5-vinylcyclopentenones, **7** and **8**, along with **5**, as metabolites of the endophytic *Streptomyces* sp. (GT-20026114) in 2005, which was obtained from the mangrove plant *Aegiceras comiculatum* [12].

**Scheme 1:**
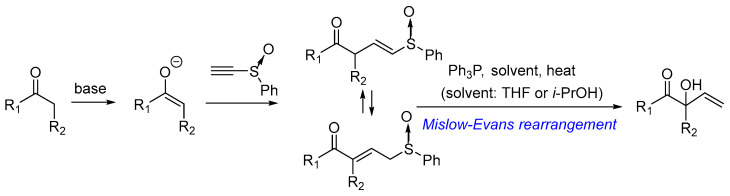
The concept of the synthesis of the α-keto vinyl carbinol from ketones by Zard.

On the other hand, synthetic studies of natural 5-hydroxy-5-vinyl-2-cyclopentenones are rare so far. To the best of our knowledge, only one example exists: the racemic synthesis of 5-hydroxy-3-methoxy-5-vinylcyclopentenone (**1**) via the acid-catalyzed ring expansion of cyclobutenol derivatives reported by Liebeskind [13]. During our continuous study on the synthesis of bioactive natural products, focusing on highly functionalized small molecules [14,15], we were interested in the synthesis of a series of natural 5-hydroxy-5-vinylcyclopentenone-type natural products. In 2013, Zard et al. reported the construction of the α-keto vinyl carbinol structural moiety by the Mizlow–Evans rearrangement of the conjugate adduct from an alkynyl sulfoxide and ketone-derived enolate, as summarized in Scheme 1 [16]. Herein, we report the racemic synthesis of a series of cyclopentenoid natural products bearing a vinyl carbinol functional group, applying this methodology to our synthetic scheme. Several reactions described in this synthetic study have been performed as microwave (MW)-assisted reactions.

## 2. Results and Discussions

**Scheme 2:**
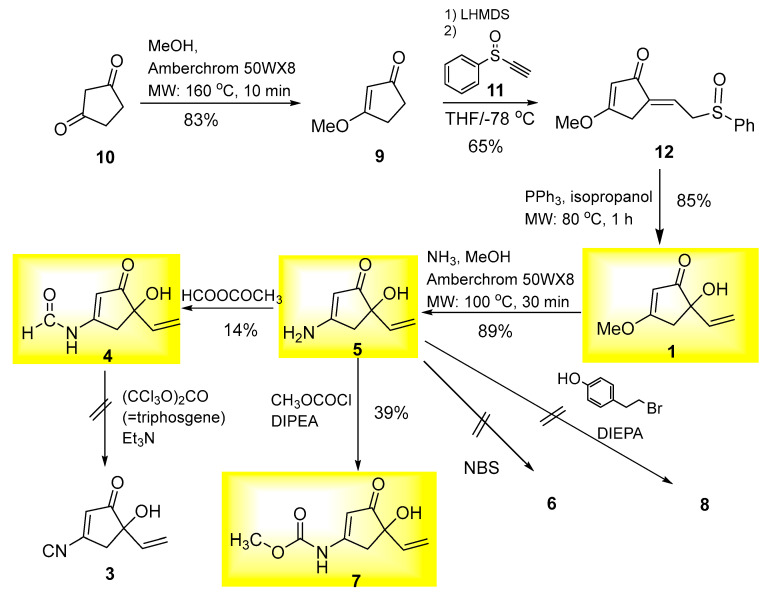
Synthesis of 5-hydroxy-5-vinylcyclopentenone-type natural products **1**, **4**, **5**, and **7**.

The target molecules **1**, **4**, **5**, and **7** were synthesized as summarized in Scheme 2. Enone **9**, derived from commercially available cyclopentan-1,3-dione (**10**) [17], was treated with lithium hexamethyldisilazide in THF at −78 °C to generate the corresponding enolate, which was then reacted with ethynyl phenyl sulfoxide (**11**) [18,19] to afford the desired conjugate adduct (*E*)-**12** (Figures SI-1 and SI-2) in 65% yield. The geometry around the exo-methylene double bond was confirmed by the NOESY cross-peaks between the two H-4 signals and the methylene signals adjacent to the sulfoxide moiety (Figure SI-4). Subsequent MW irradiation of an isopropanol solution of (*E*)-**12** at 80 °C for 1 h with triphenylphoshine successively gave the first target **1** (Figures SI-5 and SI-6) in 85% yield. The same reaction at a lower temperature (60 °C) gave a poor yield of **1** (3%). Extending the reaction time to 2 h did not improve the reaction yield (76%) and shortened the reaction time (30 min), thereby lowering the chemical yield of **1** (69% yield).

The second target, myrothenone B (**5**) (Figures SI-7, SI-8, SI-9 and SI-10), was easily prepared from **1** by a MW reaction (100 °C, 30 min) in methanolic ammonia with the Amberchrom^®^ 50WX8 in 89% yield [20]. Our attempt at the *N*-formylation of **5** into myrothenone A **4** with formic acid and *N,N*′-dicyclohexylcarbo-diimide (DCC) was unsuccessful, whereas it was previously reported in the literature [11]. Alternatively, the reaction of **5** with in situ-generated formic acetic anhydride yielded **4** (Figures SI-11 and SI-12) in 14% yield. Next, the dehydration of **4** using triphosgene was examined. However, we did not observe the formation of compound **3**. This outcome may reflect the high volatility of the target under these conditions. Further challenges regarding **3** are described in later sections. Next, we attempted to synthesize **7** and **8** from **5**, according to our early plan. The reaction of **5** with methyl chloroformate in the presence of *N*-ethyl-*N,N*-diisopropylamine (DIPEA) afforded the desired product **7** (Figures SI-13 and SI-14) in 39% yield. Meanwhile, the reaction of **5** with 4-hydroxyphenylethyl amine toward **8** did not proceed. Several other challenges in the transformation of **5** into **8** were unsuccessful.

**Scheme 3:**
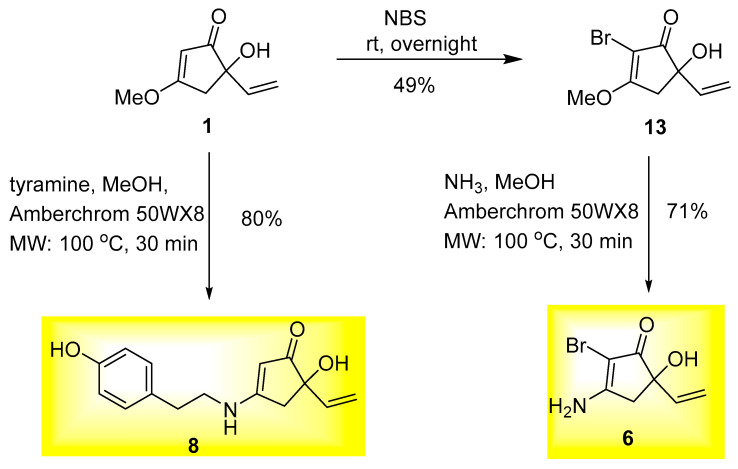
Synthesis of compounds **6** and **8** from compound **1**.

Subsequently, we focused on synthesizing 2-bromomyrothenone B (**6**). First, the bromination of **5** using an equivalent amount of *N*-bromosuccinimide (NBS) was examined. Unfortunately, the desired **6** could not be obtained. Next, the examination of the conjugate addition of the enolate derived from 2-bromo-3-methoxy-2-cyclopentenone [21] and **11** also failed. The mass spectrum of the obtained product in this experiment lacked peaks corresponding to bromine atoms. In the third trial for the synthesis of **6**, amination was set as the final step (Scheme 3). The bromination of **1** with NBS (1.0 equivalent) yielded 2-bromo-3-methoxy-5-hydroxy-5-vinyl-2-cyclopentenone (**13**) (Figures SI-17 and SI-18) in 49% yield [21], with the recovery of **1** (47%) after an overnight reaction. Compound **13** was successfully converted into the desired **6** (Figures SI-19 and SI-20) via MW-aided amination with methanolic ammonia in the presence of the Amberchrom^®^ 50WX8 in 71% yield. Encouraged by this result, the synthesis of **8** was re-examined using a similar MW reaction of **1** with methanolic tyramine to obtain the desired racemic natural product (Figures SI-15 and SI-16) in 80% yield. Hence, the synthesis of six target molecules, **1** and **4**-**8**, was accomplished.

**Scheme 4:**
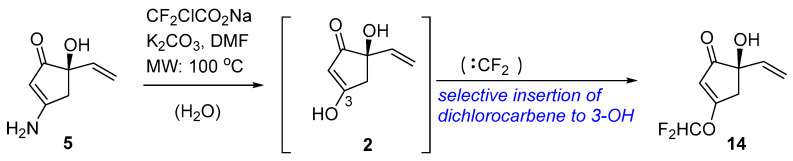
Reaction of myrothenone B (**5**) with sodium chlorofifluoroacetate.

An alternative approach to the synthesis of homothalline II (**3**) is also discussed. It has an isocyano group at C3 instead of the amino group in myrothnone B **5**. In 2020, Zhang et al. reported the transformation of an amino group into an isocyano function by difluorocarbene generated from sodium chlorodifluorochloroacetate in the presence of potassium hydroxide in *N,N*-dimethyformamide (DMF) at 100 °C [22]. Applying this reaction to compound **5** yielded 3-difluoromethoxy-5-hydroxy-5-vinyl-2-cyclopentenone (**14**) in 17% yield (recovery of **5**: 36%), as illustrated in Scheme 4, despite our best efforts to control moisture in the reaction. The structure of **14** was confirmed by detailed NMR and MS analyses (Figures SI-21 and SI-22).

## 3. Materials and Methods

### 3.1. General Methods

HRMS was performed using a JMS-700 (2) mass spectrometer (JEOL, Tokyo, Japan). NMR spectra were recorded at 27 °C on 300- and 400-MR-DD2, INOVA-500, and 600-DD2 spectrometers (Agilent Technologies, Santa Clara, CA, USA) in CDCl_3_ or acetone-d_6_ using tetramethylsilane (TMS) as an internal standard. Column chromatography was performed using silica gel (BW-127ZH) (Fuji Silysia, Tokyo, Japan). IR spectra were recorded on the IRAffinity-1S (Shimazu, Kyoto, Japan). Melting points were measured on a Yanagimoto micro-melting-point apparatus and are uncorrected. Analytical TLC was performed on precoated silica gel 60 plates (Merck & Co., Inc., Darmstadt, Germany), and the compounds were viewed by dipping the plates in an ethanol solution of phosphomolybdic acid, followed by heating. Microwave-aided reactions were performed using an Initiator^®^ microwave synthesizer (Biotage, Uppsala, Sweden). Amberchrom^®^ 50WX8-H and 2-(*p*-hydroxyphenyl)ethyl bromide were purchased from Sigma-Aldrich (St. Louis, MO, USA). *n*-BuLi in hexane, potassium carbonate, and triethylamine were purchased from Nacalai-Tesque (Kyoto, Japan). Cyclopentane-1,3-dione, hexamethyldisilazane, 30% ammonia in methanol, methyl chloroformate, and tyramine were purchased from TCI (Tokyo, Japan). Formic acid, acetic anhydride, NBS, DIPEA, triphenylphosphine, dry methanol (MeOH), dry CH_2_Cl_2_, dry DMF, dry THF, and isopropanol were purchased from Wako Pure Chemical Industries (Osaka, Japan).

### 3.2. Synthesis of 5-Hydroxy-5-vinyl-2-cyclopentenone-Type Natural Products

#### 3.2.1. (E)-3-Methoxy-5-(2-(phenylthio)ethylidene)cyclopent-2-enone (**12**)

To a solution of hexamethyldisilazane (250.7 mg, 1.5 mmol) in dry THF (1.4 mL) in a three-necked flask, *n*-BuLi (1.6 M, 0.95 mL, 1.5 mmol) was added at −78 °C under an argon atmosphere. After stirring for 30 min, a solution of enone **9** (166 mg, 1.5 mmol) in THF (1.4 mL) was added to the reaction flask at a constant temperature. After 1 h, a solution of ethynyl phenyl sulfoxide (**11**) (244 mg, 1.6 mmol) in dry THF (8.3 mL) was added to the reaction mixture, and the mixture was stirred at −78 °C for another 1 h. The reaction was quenched by the addition of saturated NH_4_Cl aq (~ 20 mL) and then extracted with EtOAc (30 mL × 3 times). The combined organic extract was dried over MgSO_4_, filtered, and evaporated under reduced pressure to obtain a crude residue, which was purified by column chromatography (eluent: EtOAc:hexane = 3:1) to afford adduct **12** (267 mg, 65%); Colorless oil; Rf; 0.1 (EtOAc:hexane = 3:1); IR (ATR): υ_max_ 1070 (S=O), 1550 (C=C), 1700 (C=O) cm^−1^; ^1^H-NMR (400 MHz, CDCl_3_): δ 2.90 (1H, br d, *J* = 20.4 Hz, =C(OMe) C*H*HC=), 3.05 (1H, br d, *J* = 20.4 Hz, =C(OMe) CH*H*C=), 3.65 (1H, dd, *J* = 12.6, 8.3 Hz, =CHC*H*HS-), 3.65 (1H, dd, *J* = 12.5, 8.8 Hz, =CHCH*H*S-), 3.85 (3H, s, -OMe), 5.48 (1H, s, -COCH=), 6.13 (1H, br tt, *J* = 8.4, 1.9 Hz, -CH_2_C=C*H*CH_2_SO-), 7.50-7.54 (3H, m, Ph-H), 7.58-7.62 (2H, m, Ph-H); ^13^C-NMR (100 MHz, CDCl_3_): δ 32.1, 57.3, 58.6, 105.8, 117.6, 124.2, 129.2, 131.5, 141.6, 142.5, 186.8; HRMS *m*/*z* calcd for C_14_H_14_O_3_S (M^+^) 262.0664, Found 262.0661.

#### 3.2.2. 5-Hydroxy-3-methoxy-5-vinylcyclopent-2-en-1-one (**1**)

Triphenylphosphine (670 mg, 2 mmol) was added to a solution of sulfoxide **12** (316 mg, 1.3 mmol) in isopropanol (9 mL) in a MW vial. The reaction vial was sealed and heated under MW irradiation at 80 °C for 1 h. After cooling, the reaction vial was opened and the reaction mixture was concentrated under reduced pressure to obtain a crude mixture, which was purified by silica gel column chromatography (eluent; EtOAc:hexane = 1:1) to afford **1** (166 mg, 85%); **1**: colorless crystals (EtOAc-hexane); mp: 57–60 °C: Rf; 0.38 (EtOAc:hexane = 3:1); IR (ATR): υ_max_ 1583 (C=C), 1696 (C=O), 3400 (OH) cm^−1^; ^1^H-NMR (600 MHz, CDCl_3_): δ 2.79 (1H, dd, *J* = 17.6, 1.2 Hz, =C(OMe)C*H*H_Cq_-), 2.85 (1H, dd, *J* = 17.6, 1.2 Hz, =C(OMe)CH*H*Cq), 3.07 (1H, br s, -OH), 3.91 (3H, s, -OMe), 5.23 (1H, dd, *J* = 10.8, 0.9 Hz, -CH=CHH), 5.32 (1H, t, *J* = 1.2 Hz, CO-C*H*=Cq), 5.42 (1H, dd, *J* = 17.2, 0.6 Hz, -CH=CHH), 5.87 (1H, dd, *J* = 17.2, 10.5 Hz, -CqCH=CH_2_); ^13^C-NMR (150 MHz, CDCl_3_): δ 42.7, 58.9, 78.7, 100.9, 114.8, 138.2, 188.6, 204.5; HRMS *m*/*z* calcd for C_8_H_10_O_3_ (M^+^) 154.0630, Found 154.0630.

#### 3.2.3. 3-Amino-5-hydroxy-5-vinylcyclopent-2-en-1-one, Myrothenone B (**5**)

To a solution of **1** (76.2 mg, 0.49 mmol) in 30% NH_3_/MeOH (14 mL) in a MW vial, Amberchrom^®^ 50WX8 (105 mg) was added. The reaction vial was sealed and heated under MW irradiation at 100 °C for 30 min. After cooling, the reaction vial was opened and the reaction mixture was evaporated under reduced pressure, and the concentrated solution was absorbed to ISOLUTE^®^ HM-N (diatomaceous earth), which was applied to purification by silica gel column chromatography (eluent; EtOAc: hexane = 3:1), affording **5** (61.3 mg, 89%); **5**: Colorless crystals (MeOH); mp: 87–89 °C: Rf; 0.69 (EtOAc); IR (ATR): υ_max_ 1604 (C=C), 1683 (C=O), 3251 (NH), 3400 (OH) cm^−1^; ^1^H-NMR (600 MHz, MeOH-d_4_): δ_,_ 2.68 (1H, dd, *J* = 17.3, 0.9 Hz, =C(OMe)C*H*H_Cq_-), 2.83 (1H, dd, *J* = 17.3, 0.9 Hz, =C(OMe)CH*H*Cq), 4.58 (1H, br s, -OH), 4.99 (1H, br t, *J* = 0.9 Hz, COCH=Cq), 5.15 (1H, dd, *J* = 10.6, 1.2 Hz, -CH=CHH), 5.36 (1H, dd, *J* = 17.3, 1.5 Hz, -CH=CHH), 5.89 (1H, dd, *J* = 17.3, 10.8 Hz, -CqCH=CH_2_); ^13^C-NMR (125 MHz, MeOH-d_4_): δ 44.2, 80.0, 97.8, 114.2, 141.2, 179.3, 204.7; HRMS *m*/*z* calcd for C_7_H_9_NO_2_ (M^+^) 139.0633, Found 139.0631.

#### 3.2.4. *N*-(4-Hydroxy-3-oxo-4-vinylcyclopent-1-en-1-yl)formamide, Myrothenone A (**4**)

Formic acid (0.6 mL, 16 mmol) and acetic anhydride (1.2 mL, 13 mmol) in a sealed MW vial were heated under MW irradiation at 65 °C for 45 min to prepare acetic formic anhydride. The obtained anhydride was added to a solution of **5** (31.8 mg, 0.21 mmol) under an argon atmosphere. After stirring overnight at room temperature, the mixture was poured into aqueous NaHCO_3_ and extracted with EtOAc three times. The combined organic layer was dried over MgSO_4_, filtered, and evaporated to obtain a crude residue, which was purified by silica gel column chromatography (eluent; EtOAc:hexane = 3:1) to afford **4** (5.4 mg, 14%) with the recovery of **5** (17.5 mg, 55%); **4:** Colorless oil: Rf; 0.5 (EtOAc:hexane = 3:1); IR (ATR): υ_max_ 1518 (C=C), 1696 (C=O), 1719 (C=O), 3210 (NH), 3346 (OH); ^1^H-NMR (600 MHz, MeOH-d_4_): δ 2.68 (1H, dd, *J* = 17.3, 0.8 Hz, =C(OMe)CHH_Cq_-), 2.83 (1H, dd, *J* = 17.3, 0.9 Hz, =C(OMe)CH*H*Cq), 4.58 (1H, br s, -OH), 4.98 (1H, t, *J* = 0.9 Hz, COCH=CqCH_2_-), 5.15 (1H, dd, *J* = 10.6, 1.2 Hz, -CH=CHH), 5.36 (1 H, dd, *J* = 17.3, 1.2 Hz, -CH=CHH), 5.89 (1H, dd, *J* = 17.3, 10.8 Hz, -CqCH=CH_2_)_,_ 8.08 (1H, s, major-NHC*H*O); ^13^C-NMR (150 MHz, MeOH-d_4_): δ, 44.2, 80.0, 97.8, 114.2 (115.1 minor)*, 141.2 (139.8 minor)*, 163.3, 179.3, 204.7. * Compound **4** is too unstable to isomerize easily in NMR spectra, as noted in the literature [11], or to observe minor signals during the measuring of ^13^C-NMR spectrum; HREIMS *m*/*z* calcd for C_8_H_9_NO_3_ (M^+^) 167.0582, Found 167.0583.

#### 3.2.5. Methyl (4-hydroxy-3-oxo-4-vinylcyclopent-1-en-1-yl)carbamate (**7**)

To a solution of **5** (105.5 mg, 0.76 mmol) in pyridine (1.0 mL), N-ethyldiisopropylamine (0.26 mL, 1.5 mmol) was added at room temperature. After stirring for 15 min, methyl chloroformate (0.14 mL, 1.5 mmol) was added to the reaction mixture. After stirring overnight at room temperature, the reaction mixture was concentrated under reduced pressure to obtain a crude residue, which was purified by silica gel column chromatography (eluent; EtOAc: hexane = 1:1 to EtOAc, then MeOH) to afford **7** (58.1 mg, 39%); **4**: Colorless crystals (MeOH); mp: 149–150 °C; Rf; 0.19 (EtOAc:hexane = 3:1); IR (ATR): υ_max_ 1598 (C=C), 1684 (CO), 1759 (C=O), 3250 (OH) cm^−1^; ^1^H-NMR (600 MHz, MeOH-d_4_): δ 2.82 (1H, dd, *J* = 17.9, 1.1 Hz, =CqCH*H*Cq-), 2.99 (1H, dd, *J* = 17.9, 0.9 Hz, =CqC*H*HCq-), 3.79 (3H, s, -CO_2_Me), 5.19 (1H, dd, *J* = 10.5, 1.2 Hz, -CH=CHH), 5.38 (1H, dd, *J* = 17.3, 1.1 Hz, -CH=CHH), 5.87 (1H, dd, *J* = 17.3, 10.5 Hz, CqCH=CH_2_)_,_ 6.05 (1H, br s, COCH=Cq); ^13^C-NMR (125 MHz, MeOH-d_4_): δ 44.6, 53.6, 78.3, 108.3, 114.9, 140.1, 154.9, 169.6, 207.9; HREIMS *m*/*z* calcd for C_9_H_11_NO_4_ (M^+^) 197.0688, Found 197.0688.

#### 3.2.6. 5-Hydroxy-3-((4-hydroxyphenethyl)amino)-5-vinylcyclopent-2-en-1-one (**8**)

A sealed vial containing a solution of **1** (41.1 mg, 0.27 mmol) in MeOH (1.0 mL), tyramine (73.8 mg, 0.54 mmol), and Amberchrom^®^ 50WX8 (10.0 mg) was heated under MW irradiation at 100 °C for 30 min. After cooling, the ingredients were concentrated under reduced pressure to obtain a crude residue, which was purified by column chromatography (eluent; EtOAc:hexane = 2:1 to EtOAc) to afford **8** (55.3 mg, 80%) with the recovery of starting **1** (3.6 mg, 9%); **8**: Colorless crystals (MeOH); mp: 78–81 °C: Rf; 0.06 (EtOAc); IR (ATR): υ_max_ 1559 (C=C), 3250 (OH) cm^−1^; ^1^H-NMR (600 MHz, MeOH-d_4_): δ 2.66 (1H, d, *J* = 16.7 Hz), 2.78 (1H, d, *J* = 16.7 Hz), 2.79 (2H, td, *J* = 7.1, 3.5 Hz), 3.40 (2H, t, *J* = 7.1 Hz), 4.95 (1H, s), 5.14 (1H, dd, *J* = 10.6, 1.1 Hz), 5.33 (1H, dd, *J* = 17.3, 1.2 Hz)_,_ 5.85 (1H, dd, *J* = 17.3, 10.9 Hz), 6.71 (2H, br d, *J* = 8.5 Hz), 7.04 (2H, br d, *J* = 8.5 Hz); ^13^C-NMR (150 MHz, MeOH-d_4_): δ 35.0, 44.2, 47.5, 79.4, 95.3, 114.3, 116.4, 130.6, 130.80, 130.81, 141.2, 157.2, 176.2, 204.2; HREIMS *m*/*z* calcd for C_15_H_18_NO_3_ ([M+H]^+^) 260.1287, Found 260.1281.

#### 3.2.7. 2-Bromo-5-hydroxy-3-methoxy-5-vinylcyclopent-2-en-1-one (**13**)

To a solution of enone **1** (89.8 mg, 0.58 mmol) in CH_2_Cl_2_ (10 mL), NBS (104 mg, 0.58 mmol) was added at 0 °C. After stirring overnight at room temperature, the solvent was removed under reduced pressure to obtain a crude residue, which was purified by column chromatography (eluent: EtOAc:hexane = 3:1) to afford **13** (67.0 mg, 49%) with the recovery of **1** (41.8 mg, 47%); **13**: Colorless crystals (EtOAc-hexane); mp: 125–127 °C; Rf; 0.47 (EtOAc:hexane = 3:1); IR (ATR): υ_max_ 3400 (OH), 1698 (C=O), 1586 (C=C) cm^−1^; ^1^H-NMR (600 MHz, CDCl_3_) δ 2.85 (1H, s, -OH), 2.94 (1H, d, *J* = 17.0 Hz, H-4), 2.97 (1H, d, *J* = 17.0 Hz, H-4′), 4.12 (3H, s, OMe), 5.30 (1H, br d, *J* = 10.5 Hz, -CH=CHH), 5.46 (1H, br d, *J* = 16.7 Hz, -CH=CHH), 5.86 (1H, dd, *J* = 17.3, 10.5 Hz, -C*H*=CH_2_); ^13^C-NMR (150 MHz, CDCl_3_) δ 41.0, 50.9, 58.3, 95.4, 115.9, 137.3, 182.5, 197.5; HRFABMS *m*/*z* calcd for C_8_H_10_^79^BrO_3_ (M+H)^+^ 232.9813, found 232.9815.

#### 3.2.8. 3-Amino-2-bromo-5-hydroxy-5-vinylcyclopent-2-en-1-one (**6**) (Bromomyrothenone B)

To a solution of **13** (61.0 mg, 0.26 mmol) in 30% NH_3_/MeOH (2.0 mL, 4.0 mmol) in a MW vial, Amberchrom ^®^ 50WX8 (25 mg) was added. The reaction vial was sealed and heated under MW irradiation at 100 °C for 30 min. After cooling, the reaction vial was opened and the reaction mixture was evaporated under reduced pressure to give a crude residue, which was purified by column chromatography (eluent: EtOAc:hexane = 3:1 to EtOAc) to afford **6** (40.5 mg, 71%); **6**: Colorless crystals (MeOH); dec: 180–182 °C; Rf; 0.19 (EtOAc: hexane = 3:1); IR (ATR) υ_max_ 1559 (C=C), 1719 (C=O), 3200 (NH), 3337 (OH) cm^−1^; ^1^H-NMR (600 MHz, MeOH-d_4_) δ 2.73 (1H, d, *J* = 17.0 Hz, H-4), 2.93 (1H, d, *J* = 17.0 Hz, H-4′), 4.58 (2H, br s, -NH_2_), 5.18 (1H, dd, *J* = 10.6, 1.2 Hz, -CH=CHH), 5.37 (1H, dd, *J* = 17.3, 1.2 Hz, -CH=CHH), 5.88 (1H, dd, *J* = 17.3, 10.6 Hz, -C*H*=CH_2_); ^13^C-NMR (150 MHz, MeOH-d_4_) δ 43.3, 78.5, 88.0, 114.8, 140.5, 173.3, 197.1; ^1^H-NMR (600 MHz, DMSO-d_6_) δ 2.56 (1H, d, *J* = 17.0 Hz, H-4), 2.79 (1H, d, *J* = 17.0 Hz, H-4′), 5.07 (1H, dd, *J* = 10.6, 1.6 Hz, -CH=CHH), 5.26 (1H, dd, *J* = 17.3, 1.6 Hz, -CH=CHH), 5.79 (1H, dd, *J* = 17.3, 10.6 Hz, -C*H*=CH_2_), 7.53 (1H, br s, -N*H*H), 8.01 (1H, br s, -NH*H*); ^13^C-NMR (150 MHz, DMSO-d_6_) δ 42.0, 76.4, 86.1, 113.2, 140.4, 169.5, 193.2; HREIMS *m*/*z* calcd for C_7_H_8_^79^BrO_2_ (M)^+^ 216.9738, found 216.9741.

#### 3.2.9. Reaction of Myrothenone B (**5**) with Difluorocarbene

To a solution of **5** (50.5 mg, 0.36 mmol) in dry DMF (5.0 mL) in a three-necked flask, potassium carbonate (100.8 mg, 0.73 mmol) and molecular sieves 4A (1 g) were added. After stirring the mixture under an argon atmosphere at room temperature for 30 min, sodium chlorodifluoroacetate (111.2 mg, 0.73 mmol) was added. The flask was heated in an oil bath at 100 °C for 4 h. After cooling, the reaction mixture was diluted with CH_2_Cl_2_ (40 mL) and washed with water (50 mL) and brine (50 mL) × 2. The organic layer was dried over MgSO_4_, filtered, and concentrated under reduced pressure to obtain a crude residue, which was purified by column chromatography (eluent: EtOAc:hexane = 1:1) to afford 3-difluoromethoxy-5-hydroxy-5-vinylcyclopent-2-en-1-one (**14**) (11.5 mg, 17%) as a colorless oil with the recovery of **5** (18.5 mg, 36%); **14**: Oil; Rf; 0.41 (EtOAc:hexane = 1:1); IR (ATR) υ_max_ 1698(C=O), 1612 (C=C), 3370 (OH) cm^−1^; ^1^H-NMR (600 MHz, CDCl_3_) δ 2.39 (1H, s, OH), 2.72 (1H, br d, *J* = 17.9 Hz, H-4), 2.75 (1H, d, *J* = 18.2 Hz, H-4′), 5.37 (1H, d, *J* = 10.5, -CH=CHH), 5.47 (1H, d, *J* = 17.3 Hz, -CH=CHH), 5.62 (1H, t, *J* = 1.4 Hz, H-2), 6.01 (1H, dd, *J* = 17.3, 10.5 Hz, -CH=CHH), 6.56 (1H, dd, ^2^*J_H-F_* = 72.5, 70.4 Hz, C*H*F_2_); ^13^C-NMR (150 MHz, CDCl_3_) δ 49.3, 77.4, 110.2, 114.9 (t, *J* = 265.8 Hz), 116.2, 137.6, 177.6, 199.6; HREIMS *m*/*z* calcd for C_8_H_8_F_2_O_3_ (M)^+^ 190.0441, found 190.0438.

## 4. Conclusions

We achieved racemic synthesis of six natural 5-hydroxy-5-vinyl-2-cyclopentenones. Compounds **5**–**8** were synthesized for the first time in this study. The key reactions for constructing 5-hydroxy-3-methoxy-5-vinyl-2-cyclopentenone were conjugate addition between the 3-methoxy-2-cyclopentenone-derived enolate and ethynyl phenyl sulfoxide, and the subsequent Mislow–Evans rearrangement. In this synthesis, many MW-aided reactions resulted in shorter and cleaner reactions.

Our efforts toward the final target **3** will continue, and a new project for the asymmetric synthesis of this type of natural product has been initiated.

## Data Availability

The original contributions presented in this study are included in the article. Further inquiries can be directed to the corresponding author.

## Supplementary Materials

The following supporting information can be downloaded at https://www.mdpi.com/article/10.3390/md23120449/s1. ^1^H-NMR spectrum of **12** in CDCl_3_ (Figure SI-1). ^13^C-NMR spectrum of compound **12** in CDCl_3_ (Figure SI-2). The COSY spectrum of compound **12** (Figure SI-3). The NOESY spectrum of compound **12** (Figure SI-4). ^1^H-NMR spectrum of compound **1** in CDCl_3_ (Figure SI-5). ^13^C-NMR spectrum of compound **1** in CDCl_3_ (Figure SI-6). ^1^H-NMR spectrum of compound **5** in MeOH-d_4_ (Figure SI-7). ^13^C-NMR spectrum of compound **5** in MeOH-d_4_ (Figure SI-8). ^1^H-NMR spectrum of compound **5** in DMSO-d_6_ (Figure SI-9). ^13^C-NMR spectrum of compound **5** in DMSO-d_6_ (Figure SI-10). ^1^H-NMR spectrum of compound **4** in MeOH-d_4_(Figure SI-11). ^13^C-NMR spectrum of compound **4** in MeOH-d_4_ (Figure SI-12). ^1^H-NMR spectrum of compound **7** in MeOH-d_4_ (Figure SI-13). ^13^C-NMR spectrum of compound **7** in MeOH-d_4_ (Figure SI-14). ^1^H-NMR spectrum of compound **8** in MeOH-d_4_ (Figure SI-15). ^13^C-NMR spectrum of compound **8** in MeOH-d_4_ (Figure SI-16). ^1^H-NMR spectrum of compound **13** in CDCl_3_ (Figure SI-17). ^13^C-NMR spectrum of compound **13** in CDCl_3_ (Figure SI-18). ^1^H-NMR spectrum of compound **6** in MeOH-d_4_ (Figure SI-19). ^13^C-NMR spectrum of compound **6** in MeOH-d_4_ (Figure SI-20). ^1^H-NMR spectrum of compound **14** in DMSO-d_6_ (Figure SI-21). ^13^C-NMR spectrum of compound **14** in DMSO-d_6_ (Figure SI-22).
