# Supplementary material for: Synthesis of 5-Hydroxy-5-vinyl-2-cyclopentenones, a Family of Rare-Type Natural Products Mostly Recovered from Marine Sources
Source: Mar Drugs. 2025 Nov 23;23(12):449. doi: 10.3390/md23120449 (PMC12734637; doi:10.3390/md23120449)

Supporting Information

Synthesis of 5-Hydroxy-5-vinyl-2-cyclopentenones, a Family of Rare-Type Natural Products Mostly Recovered from Marine Sources

Yoshihide Usami,*, Natsuki Asada, Chihiro Shizuma, Karin Negoro, Ryosuke Kawai, Sayaka Kaneda, and Noboru Hayama

Osaka Medical and Pharmaceutical University

*Corresponding author: yoshihide.usami@ompu.ac.jp

Contents

Figure SI-1. ^{1}H-NMR spectrum of compound **12** in $CDCl_3$ SI-2
Figure SI-2. ^{13}C-NMR spectrum of compound **12** in $CDCl_3$ SI-2
Figure SI-3. COSY and Figure SI-4: NOESY spectrum of compound **12** SI-3
Figure SI-5. ^{1}H-NMR spectrum of compound **1** in $CDCl_3$ SI-4
Figure SI-6. ^{13}C-NMR spectrum of compound **1** in $CDCl_3$ SI-4
Figure SI-7. ^{1}H-NMR spectrum of compound **5** in MeOH-d_4 SI-5
Figure SI-8. ^{13}C-NMR spectrum of compound **5** in MeOH-d_4 SI-5
Figure SI-9. ^{1}H-NMR spectrum of compound **5** in DMSO-d_6 SI-6
Figure SI-10. ^{13}C-NMR spectrum of compound **5** in DMSO-d_6 SI-6
Figure SI-11. ^{1}H-NMR spectrum of compound **4** in MeOH-d_4 SI-7
Figure SI-12. ^{13}C-NMR spectrum of compound **4** in MeOH-d_4 SI-7
Figure SI-13. ^{1}H-NMR spectrum of compound **7** in MeOH-d_4 SI-8
Figure SI-14. ^{13}C-NMR spectrum of compound **7** in MeOH-d_4 SI-8
Figure SI-15. ^{1}H-NMR spectrum of compound **8** in MeOH-d_4 SI-9
Figure SI-16. ^{13}C-NMR spectrum of compound **8** in MeOH-d_4 SI-9
Figure SI-17. ^{1}H-NMR spectrum of compound **13** in $CDCl_3$ SI-10
Figure SI-18. ^{13}C-NMR spectrum of compound **13** in $CDCl_3$ SI-10
Figure SI-19. ^{1}H-NMR spectrum of compound **6** in DMSO-d_6 SI-11
Figure SI-20. ^{13}C-NMR spectrum of compound **6** in DMSO-d_6 SI-11
Figure SI-21. ^{1}H-NMR spectrum of compound **14** in $CDCl_3$ SI-12
Figure SI-22. ^{13}C-NMR spectrum of compound **14** in $CDCl_3$ SI-12

Figure SI-1. ^{1}H-NMR spectrum of compound **12** (600 MHz, $CDCl_3$)

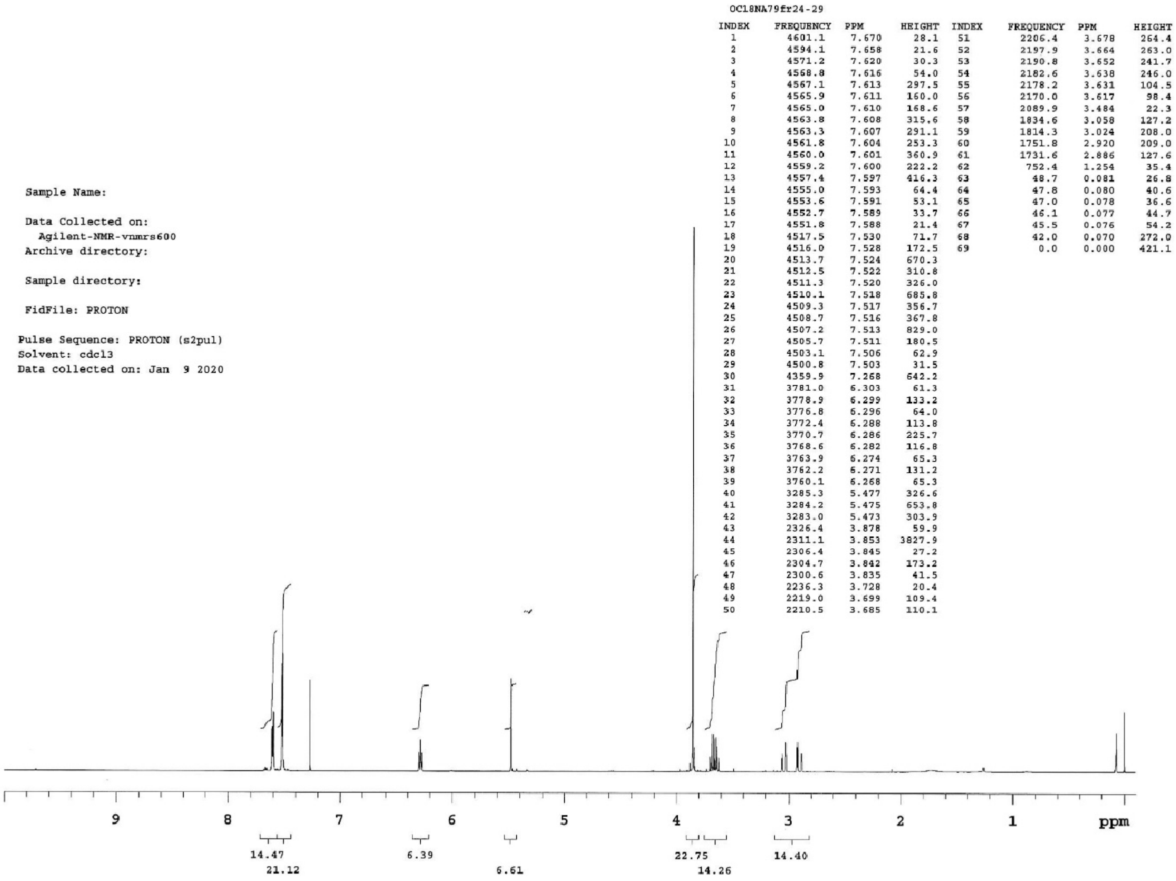

Figure SI-2: ^{13}C-NMR spectrum of compound **12** (150 MHz, $CDCl_3$)

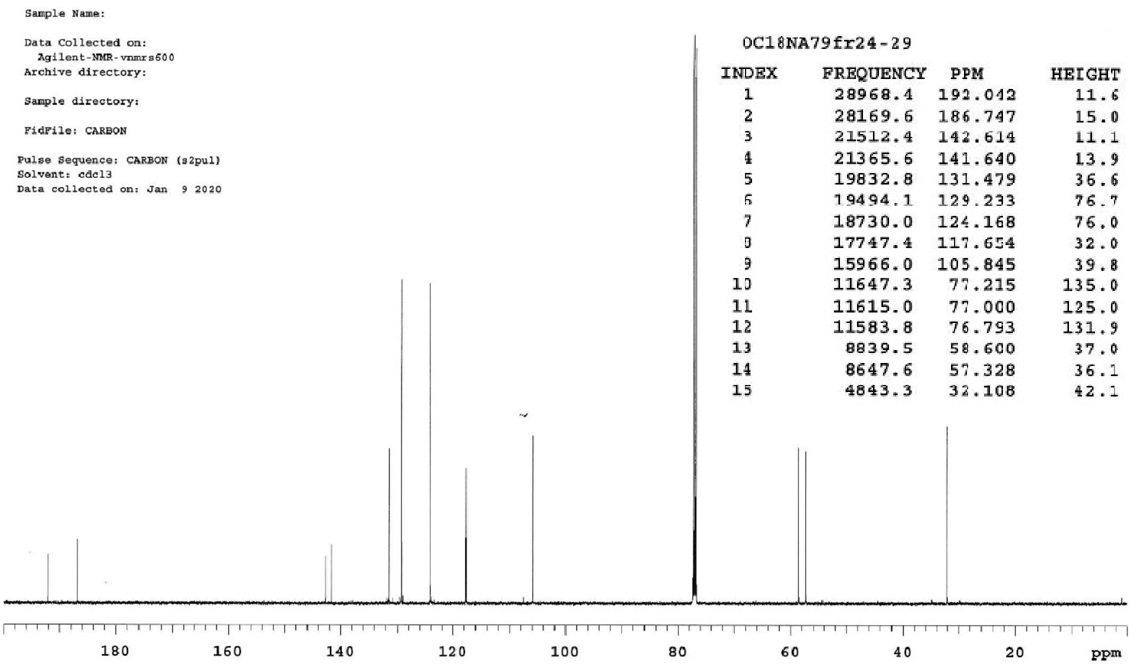

Figure SI-3: COSY spectrum of compound **12** (600 MHz, $CDCl_3$)

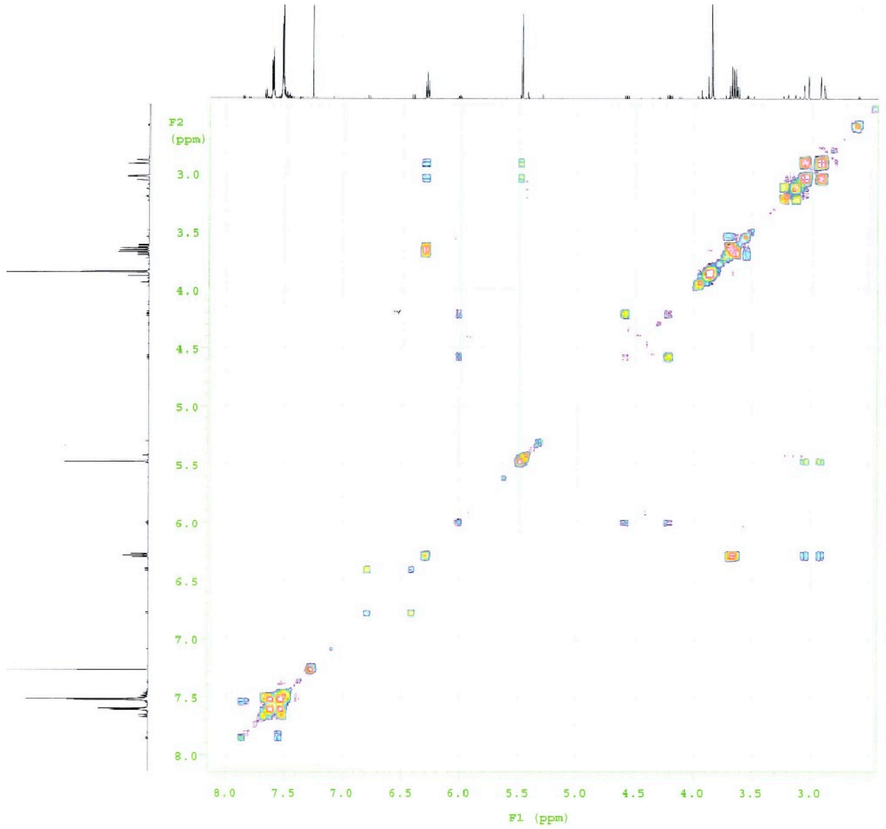

Figure SI-4: NOESY spectrum of compound **12** (600 MHz, $CDCl_3$)

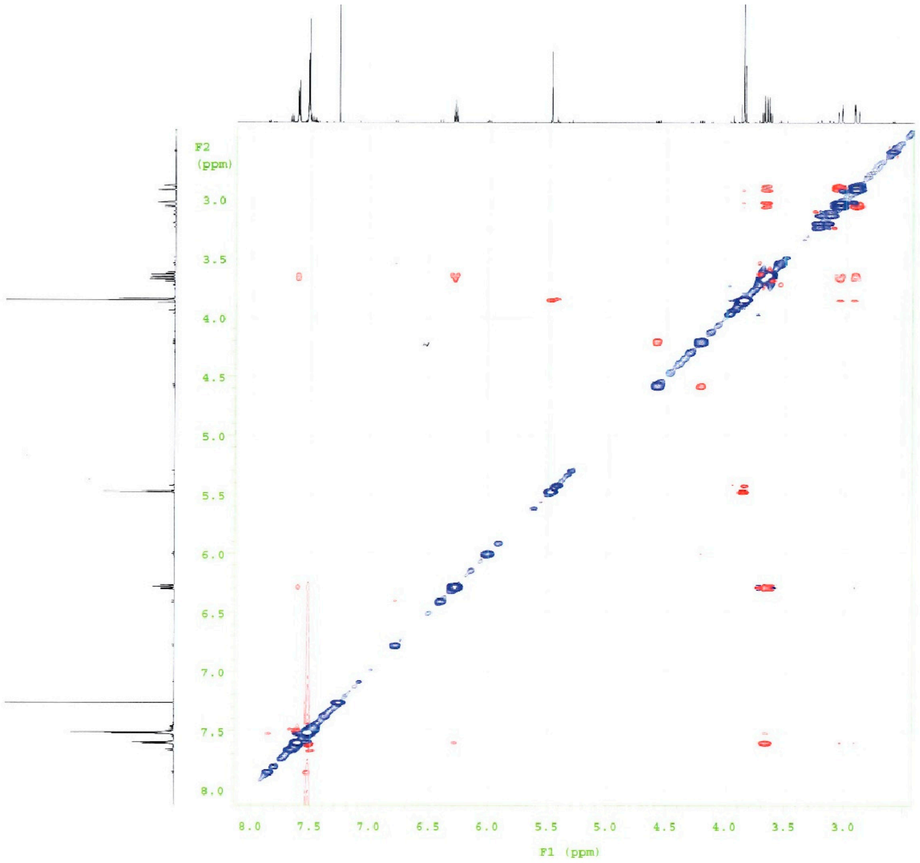

Figure SI-5: ^{1}H-NMR spectrum of compound **1** (600 MHz, $CDCl_3$)

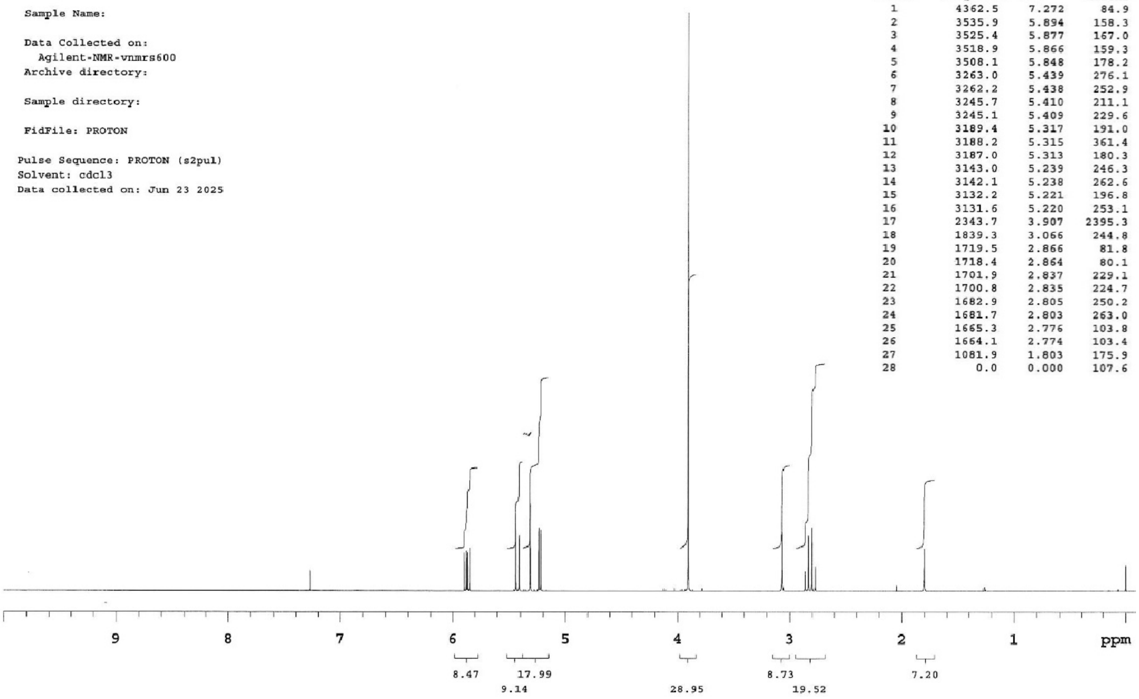

OC24KY35

INDEX	FREQUENCY	PPM	HEIGHT
1	4362.5	7.272	84.9
2	3535.9	5.894	158.3
3	3525.4	5.877	167.0
4	3518.9	5.866	159.3
5	3508.1	5.848	178.2
6	3263.0	5.439	276.1
7	3262.2	5.438	252.9
8	3245.7	5.410	211.1
9	3245.1	5.409	229.6
10	3189.4	5.317	191.0
11	3188.2	5.315	361.4
12	3187.0	5.313	180.3
13	3143.0	5.239	246.3
14	3142.1	5.238	262.6
15	3132.2	5.221	196.8
16	3131.6	5.220	253.1
17	2343.7	3.907	2395.3
18	1839.3	3.066	244.8
19	1719.5	2.866	81.8
20	1718.4	2.864	80.1
21	1701.9	2.837	229.1
22	1700.8	2.835	224.7
23	1682.9	2.805	250.2
24	1681.7	2.803	263.0
25	1665.3	2.776	103.8
26	1664.1	2.774	103.4
27	1081.9	1.803	175.9
28	0.0	0.000	107.6

Figure SI-6: ^{13}C-NMR spectrum of compound **1** (150 MHz, $CDCl_3$)

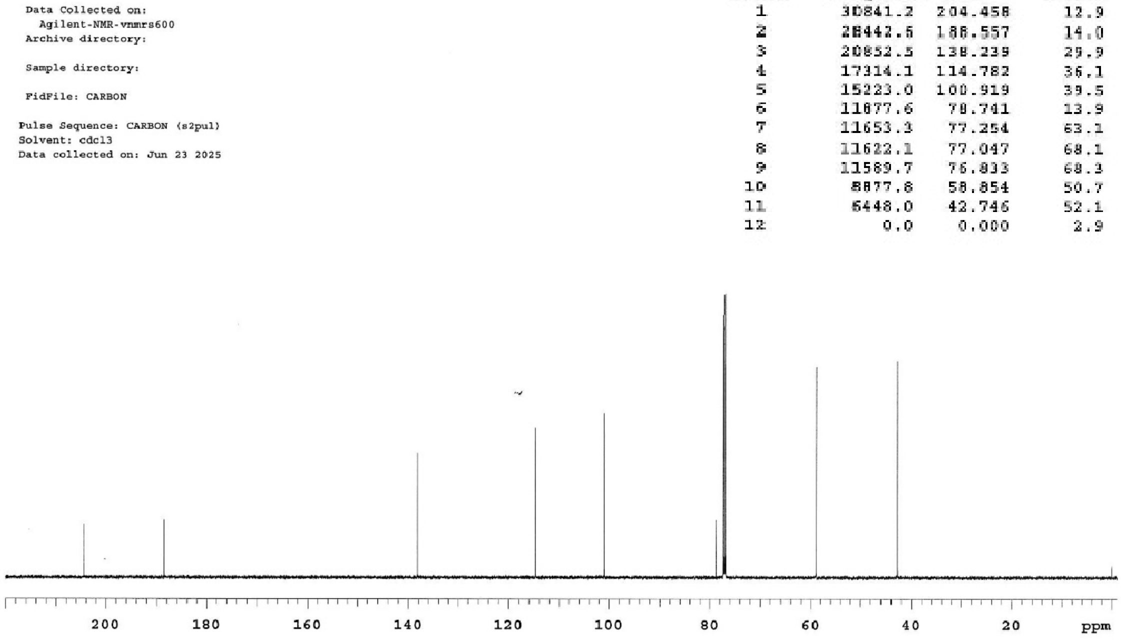

OC24KY35

INDEX	FREQUENCY	PPM	HEIGHT
1	30841.2	204.458	12.9
2	28442.6	188.557	14.0
3	20852.5	138.239	29.9
4	17314.1	114.782	36.1
5	15223.0	100.919	39.5
6	11877.6	78.741	13.9
7	11653.3	77.254	63.1
8	11622.1	77.047	68.1
9	11589.7	76.833	68.3
10	8877.6	58.854	50.7
11	6448.0	42.746	52.1
12	0.0	0.000	2.9

Figure SI-7: ^{1}H-NMR spectrum of compound **5** (600 MHz, MeOH-d$_4$)

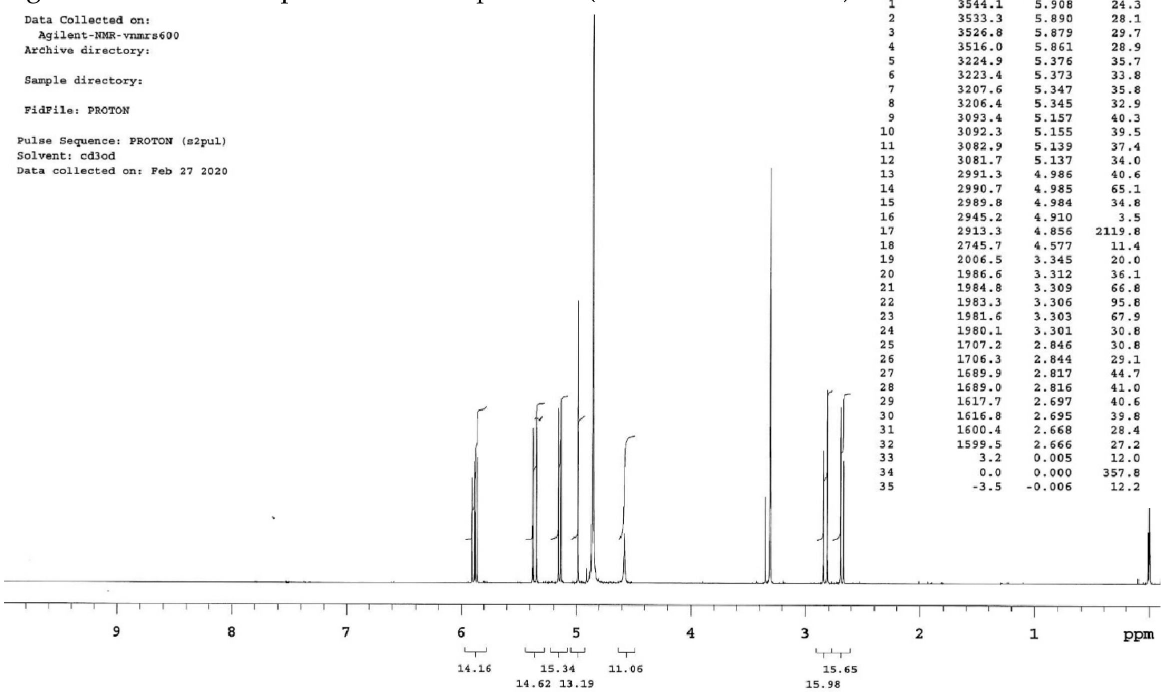

OC18NA97 fr4-7 in CD3OD

INDEX	FREQUENCY	PPM	HEIGHT
1	3544.1	5.908	24.3
2	3533.3	5.890	28.1
3	3526.8	5.879	29.7
4	3516.0	5.861	28.9
5	3224.9	5.376	35.7
6	3223.4	5.373	33.8
7	3207.6	5.347	35.8
8	3206.4	5.345	32.9
9	3093.4	5.157	40.3
10	3092.3	5.155	39.5
11	3082.9	5.139	37.4
12	3081.7	5.137	34.0
13	2991.3	4.986	40.6
14	2990.7	4.985	65.1
15	2989.8	4.984	34.8
16	2945.2	4.910	3.5
17	2913.3	4.856	2119.8
18	2745.7	4.577	11.4
19	2006.5	3.345	20.0
20	1986.6	3.312	36.1
21	1984.8	3.309	66.8
22	1983.3	3.306	95.8
23	1981.6	3.303	67.9
24	1980.1	3.301	30.8
25	1707.2	2.846	30.8
26	1706.3	2.844	29.1
27	1689.9	2.817	44.7
28	1689.0	2.816	41.0
29	1617.7	2.697	40.6
30	1616.8	2.695	39.8
31	1600.4	2.668	28.4
32	1599.5	2.666	27.2
33	3.2	0.005	12.0
34	0.0	0.000	357.8
35	-3.5	-0.006	12.2

Figure SI-8: ^{13}C-NMR spectrum of compound **5** (150 MHz, MeOH-d$_4$)

OC18NA97 fr4-7 in CD3OD

INDEX	FREQUENCY	PPM	HEIGHT
1	30880.6	204.718	16.1
2	27040.4	179.261	22.7
3	21298.7	141.197	54.0
4	17233.2	114.245	56.5
5	14758.3	97.838	49.4
6	12068.3	80.005	18.8
7	7461.8	49.467	147.8
8	7441.0	49.329	536.9
9	7419.0	49.183	912.9
10	7398.2	49.045	1223.0
11	7376.2	48.900	969.6
12	7355.4	48.762	515.9
13	7333.5	48.616	171.3
14	6669.9	44.217	54.3
15	0.0	0.000	36.4

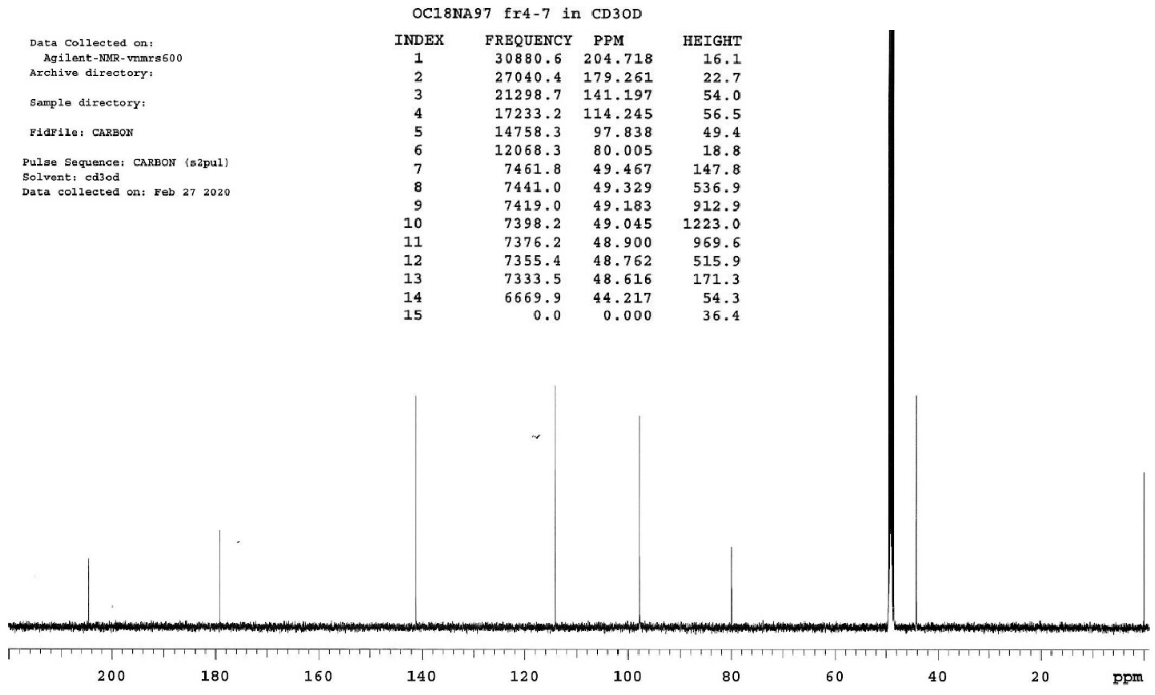

Figure SI-9: ^{1}H-NMR spectrum of compound **5** (600 MHz, DMSO-d_6)

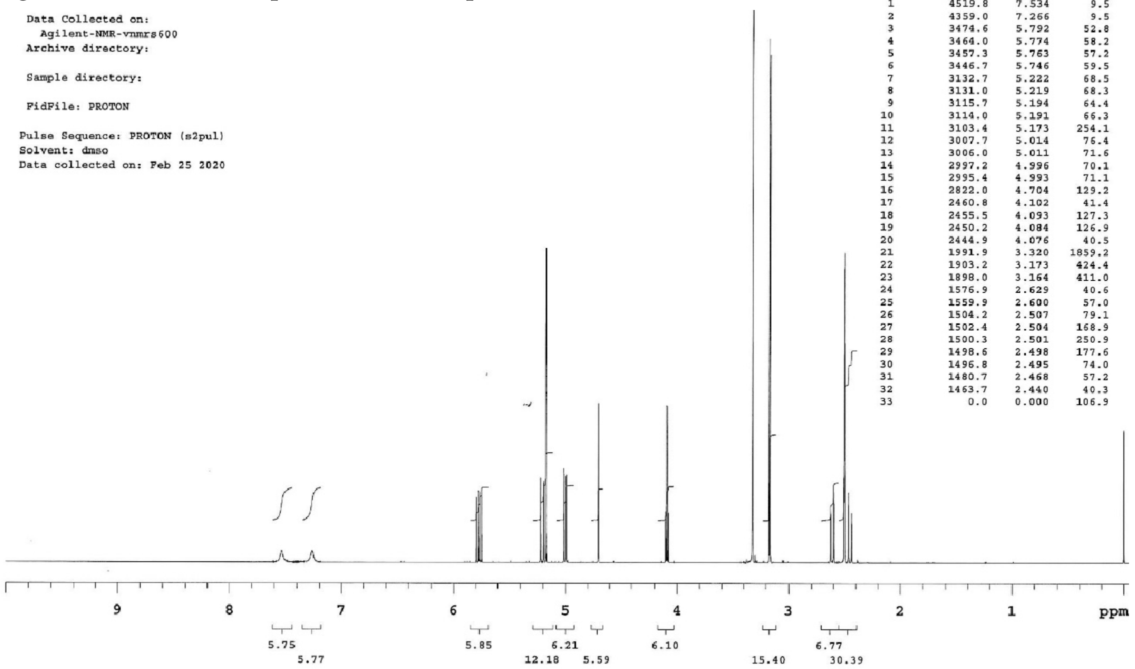

Figure SI-10: ^{13}C-NMR spectrum of compound **5** (150 MHz, DMSO-d_6)

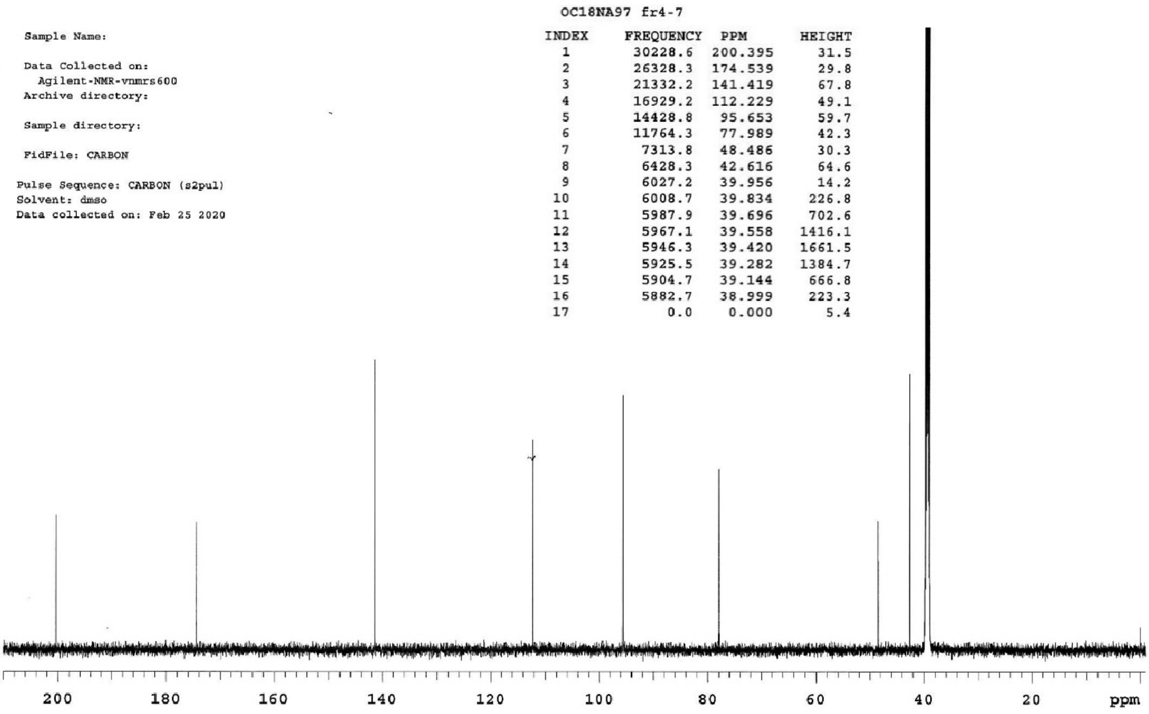

Figure SI-11: ^{1}H-NMR spectrum of compound **4** (600 MHz, MeOH-d4)

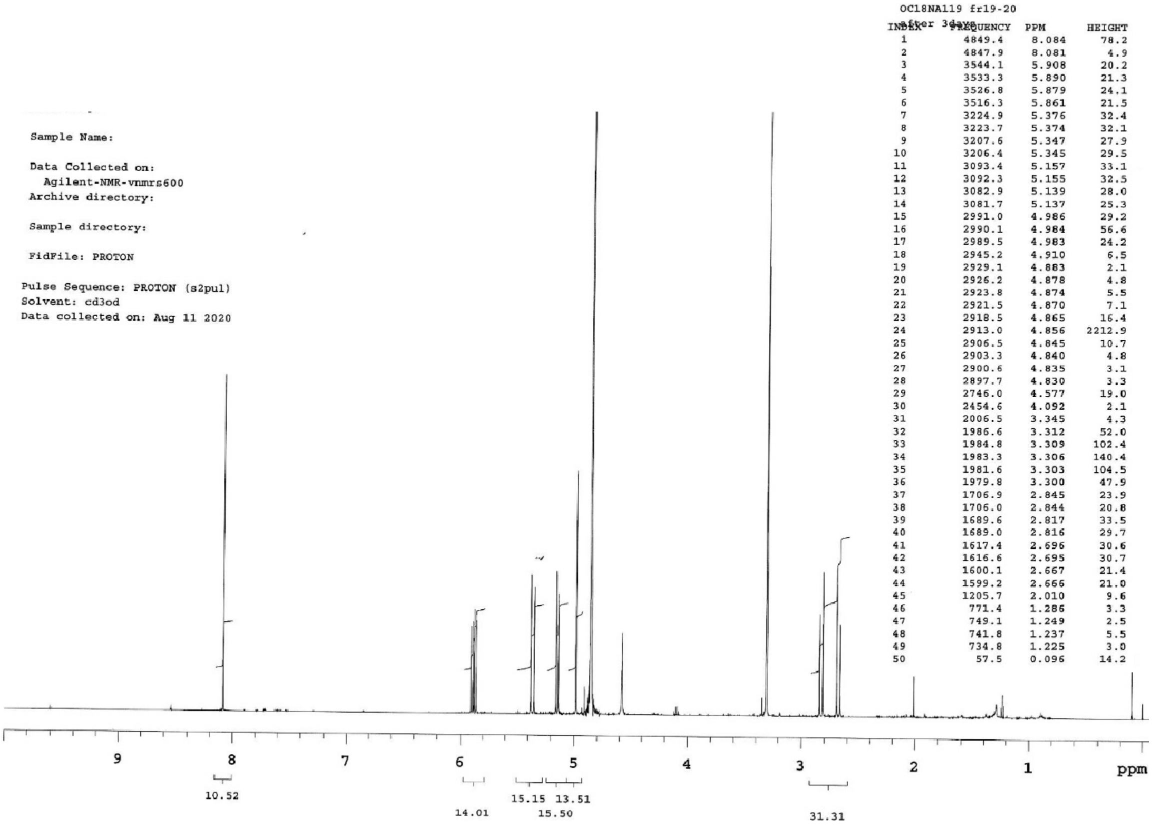

Figure SI-12: ^{13}C-NMR spectrum of compound **4** (150 MHz, MeOH-d4)

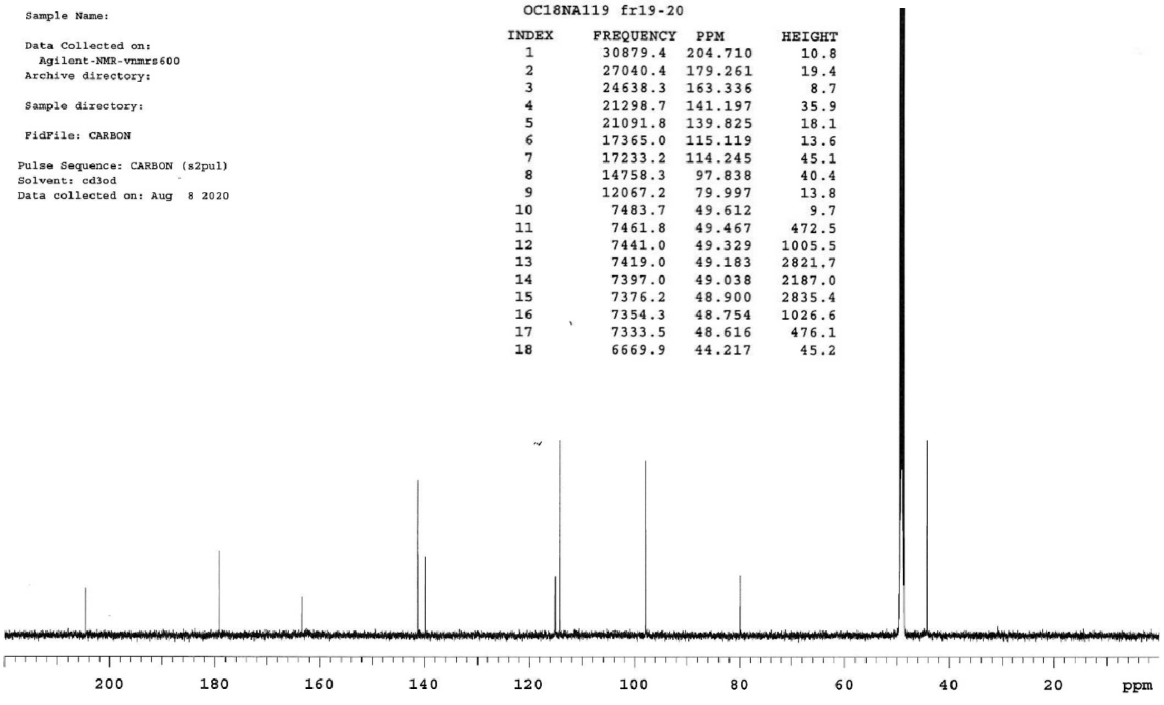

Figure SI-13: ^{1}H-NMR spectrum of compound **7** (600 MHz, MeOH-d$_4$)

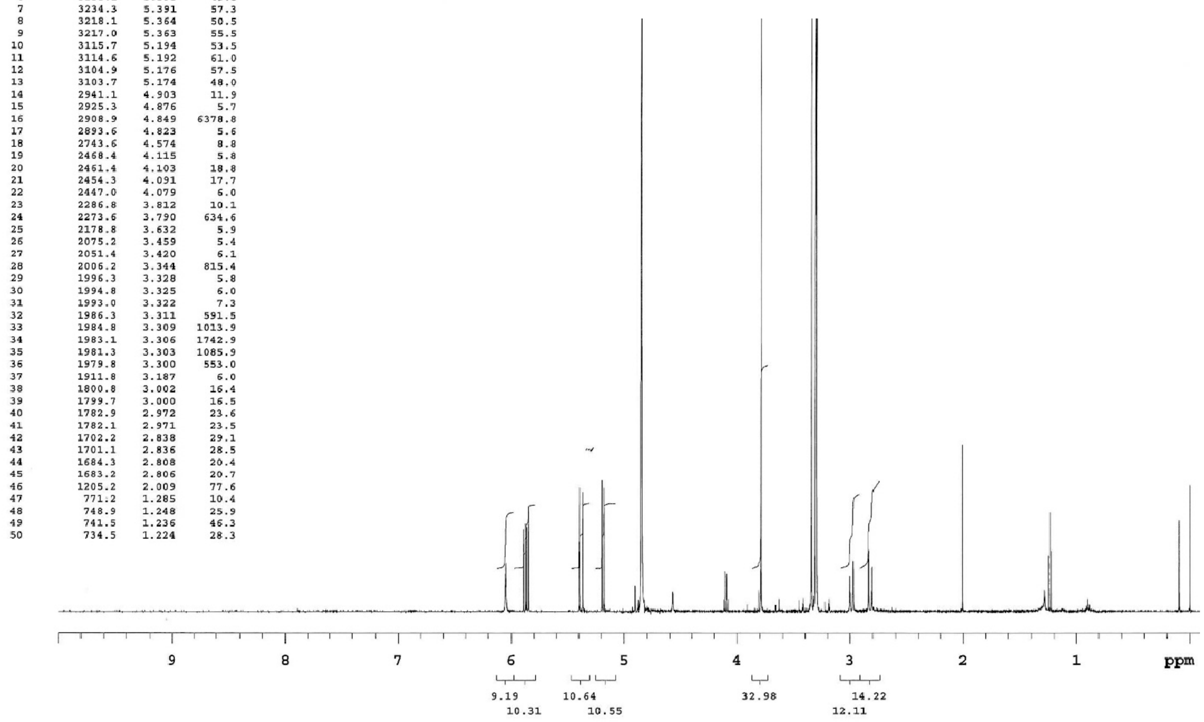

INDEX	FREQUENCY	PPM	HEIGHT
1	3629.0	6.049	22.1
2	3535.6	5.894	38.0
3	3525.1	5.876	41.0
4	3518.3	5.865	39.0
5	3507.8	5.847	46.7
6	3235.2	5.393	49.5
7	3234.3	5.391	57.3
8	3218.1	5.364	50.5
9	3217.0	5.363	55.5
10	3115.7	5.194	53.5
11	3114.6	5.192	61.0
12	3104.9	5.176	57.5
13	3103.7	5.174	48.0
14	2941.1	4.903	11.9
15	2925.3	4.876	5.7
16	2908.9	4.849	6378.8
17	2893.6	4.823	5.6
18	2743.6	4.574	8.8
19	2468.4	4.115	5.8
20	2461.4	4.103	18.8
21	2454.3	4.091	17.7
22	2447.0	4.079	6.0
23	2286.8	3.812	10.1
24	2273.6	3.790	634.6
25	2178.8	3.632	5.9
26	2075.2	3.459	5.4
27	2051.4	3.420	6.1
28	2006.2	3.344	815.4
29	1996.3	3.328	5.8
30	1994.8	3.325	6.0
31	1993.0	3.322	7.3
32	1986.3	3.311	591.5
33	1984.8	3.309	1013.9
34	1983.1	3.306	1742.9
35	1981.3	3.303	1085.9
36	1979.8	3.300	553.0
37	1911.8	3.187	6.0
38	1800.8	3.002	16.4
39	1799.7	3.000	16.5
40	1782.9	2.972	23.6
41	1782.1	2.971	23.5
42	1702.2	2.838	29.1
43	1701.1	2.836	28.5
44	1684.3	2.808	20.4
45	1683.2	2.806	20.7
46	1205.2	2.009	77.6
47	771.2	1.285	10.4
48	748.9	1.248	25.9
49	741.5	1.236	46.3
50	734.5	1.224	28.3
51	538.8	0.898	5.9
52	57.5	0.096	42.2
53	0.0	0.000	58.8

Figure SI-14: ^{13}C-NMR spectrum of compound **7** (150 MHz, MeOH-d$_4$)

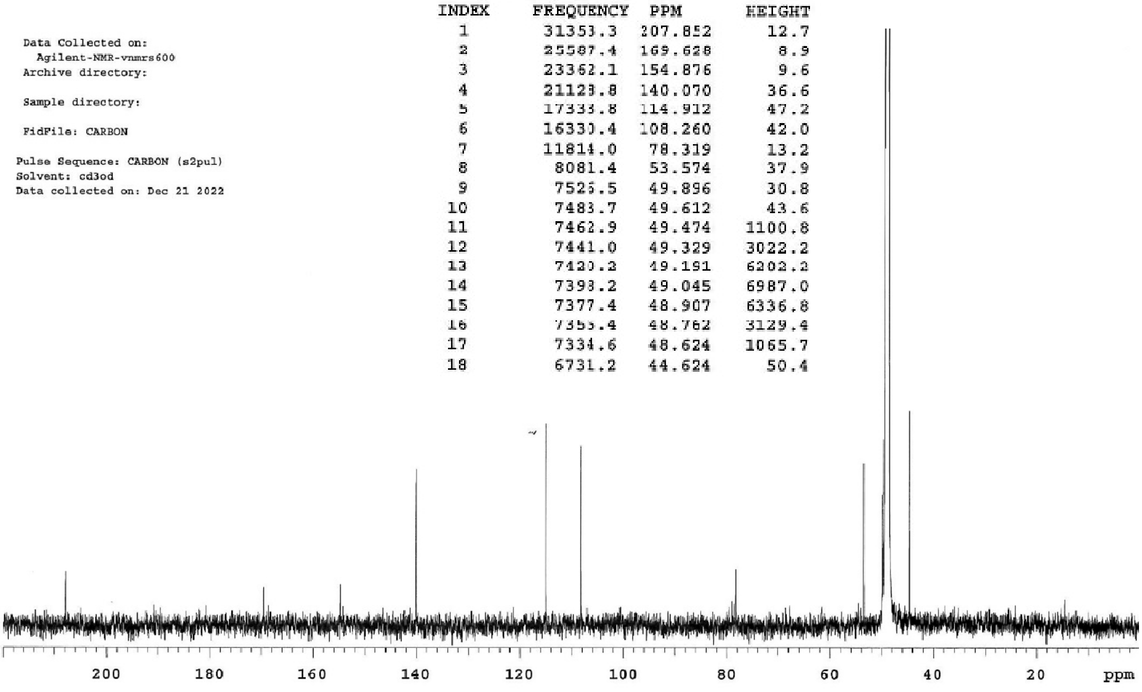

INDEX	FREQUENCY	PPM	HEIGHT
1	31353.3	207.852	12.7
2	25587.4	169.628	8.9
3	23362.1	154.876	9.6
4	21123.8	140.070	36.6
5	17333.8	114.912	47.2
6	16330.4	108.260	42.0
7	11814.0	78.319	13.2
8	8081.4	53.574	37.9
9	7526.5	49.896	30.8
10	7483.7	49.612	43.6
11	7462.9	49.474	1100.8
12	7441.0	49.329	3022.2
13	7420.2	49.191	6202.2
14	7398.2	49.045	6987.0
15	7377.4	48.907	6336.8
16	7355.4	48.762	3129.4
17	7334.6	48.624	1065.7
18	6731.2	44.624	50.4

Figure SI-15: ^{1}H-NMR spectrum of compound **8** (600 MHz, MeOH-d4)

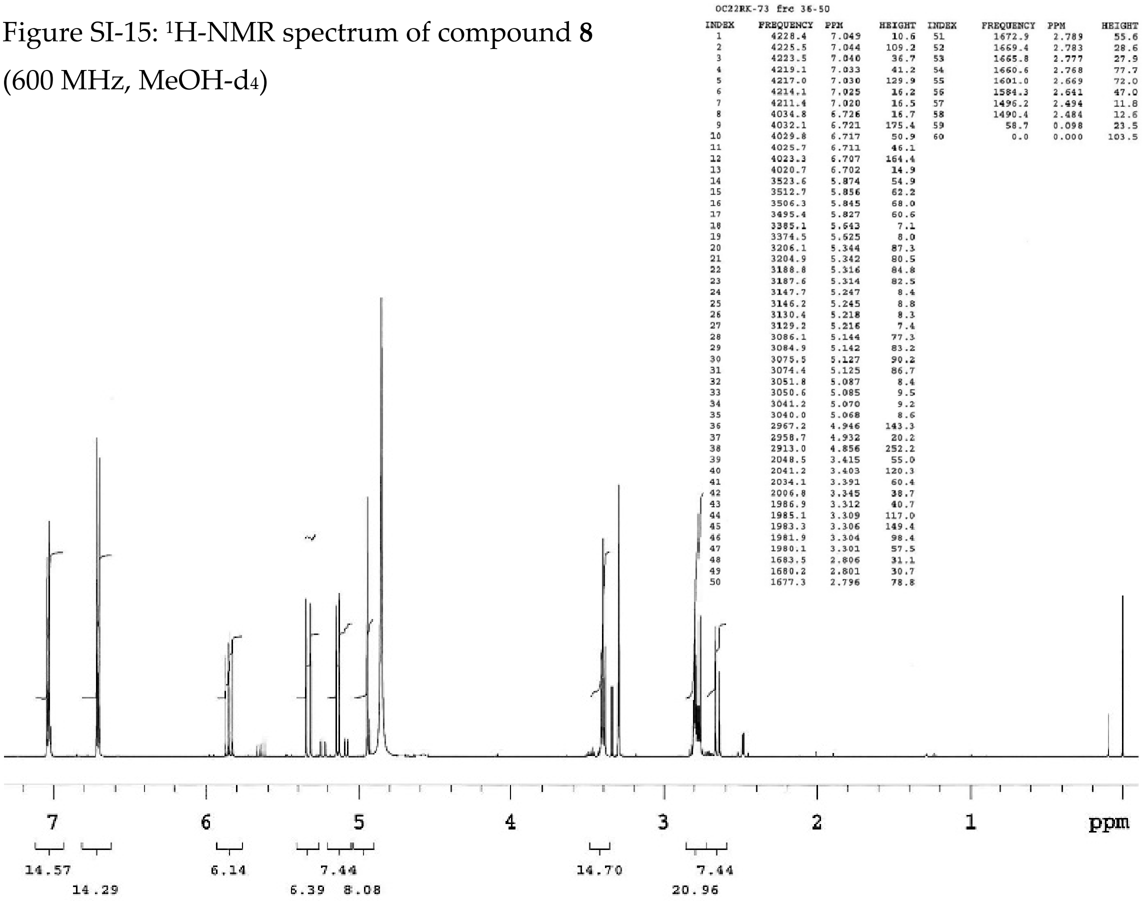

Figure SI-16: ^{13}C-NMR spectrum of compound **8** (150 MHz, MeOH-d4)

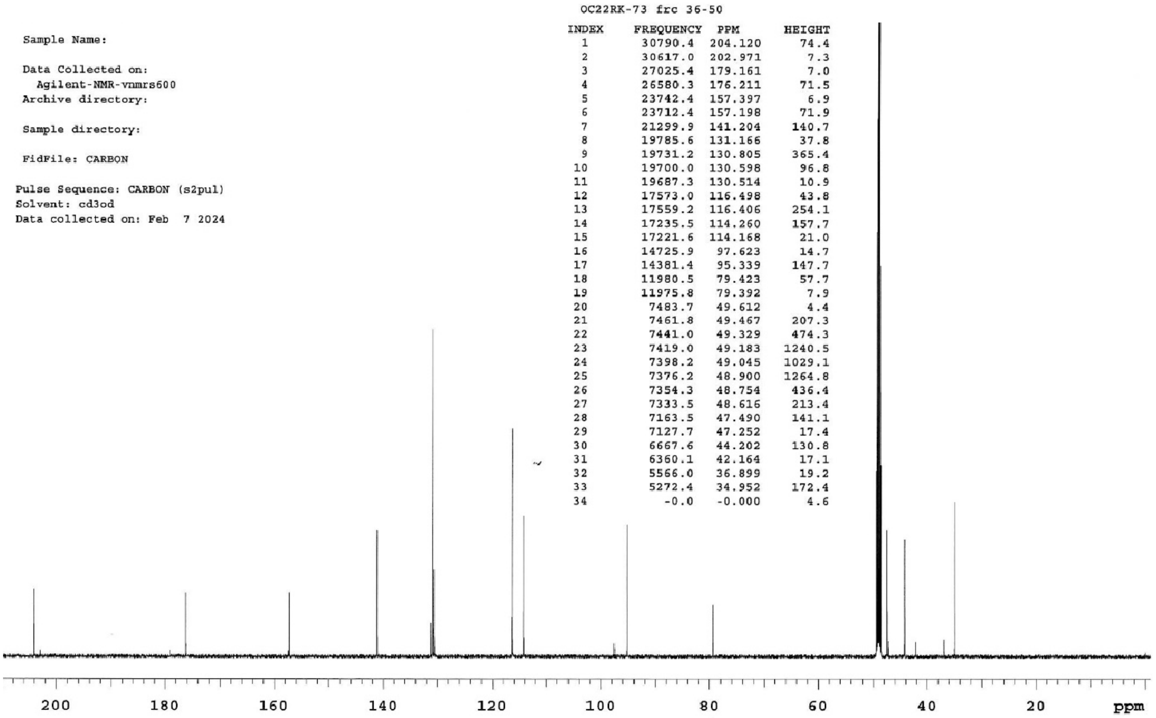

Figure SI-17: ^{1}H-NMR spectrum of compound **13** (600 MHz, $CDCl_3$)

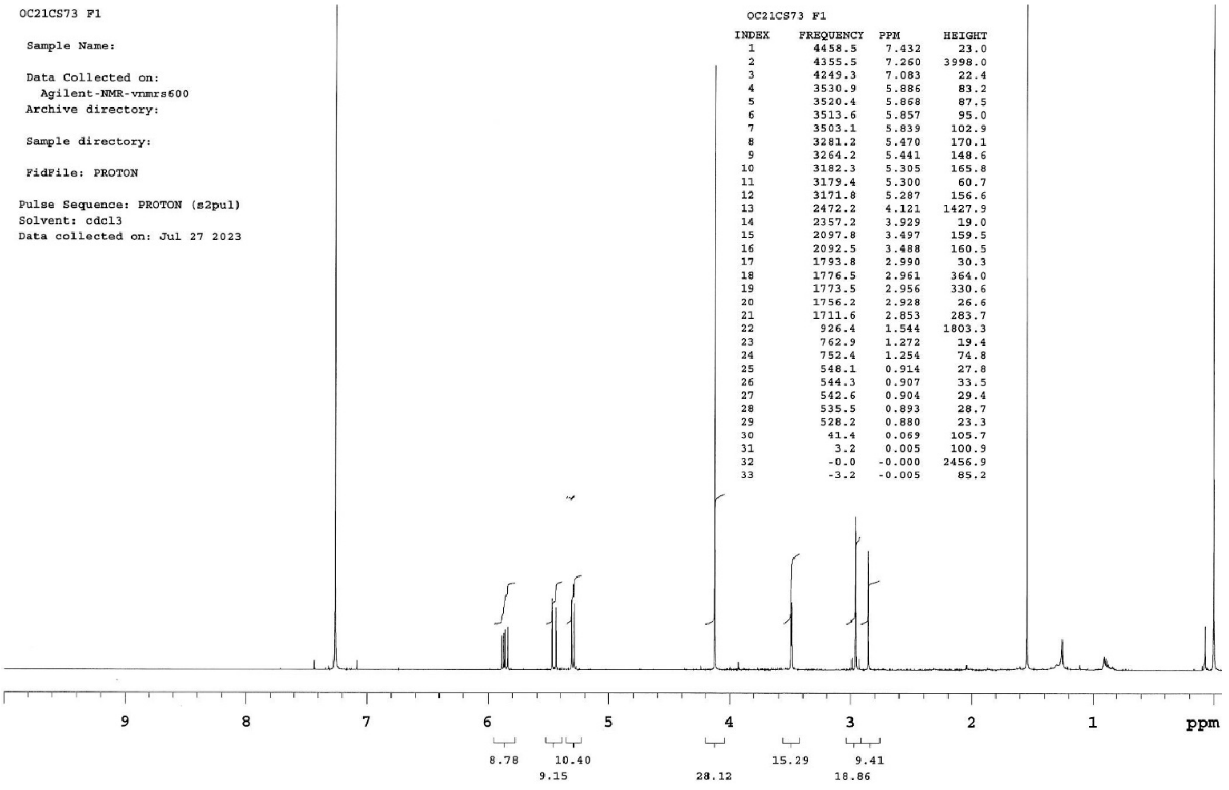

Figure SI-18: ^{13}C-NMR spectrum of compound **13** (150 MHz, $CDCl_3$)

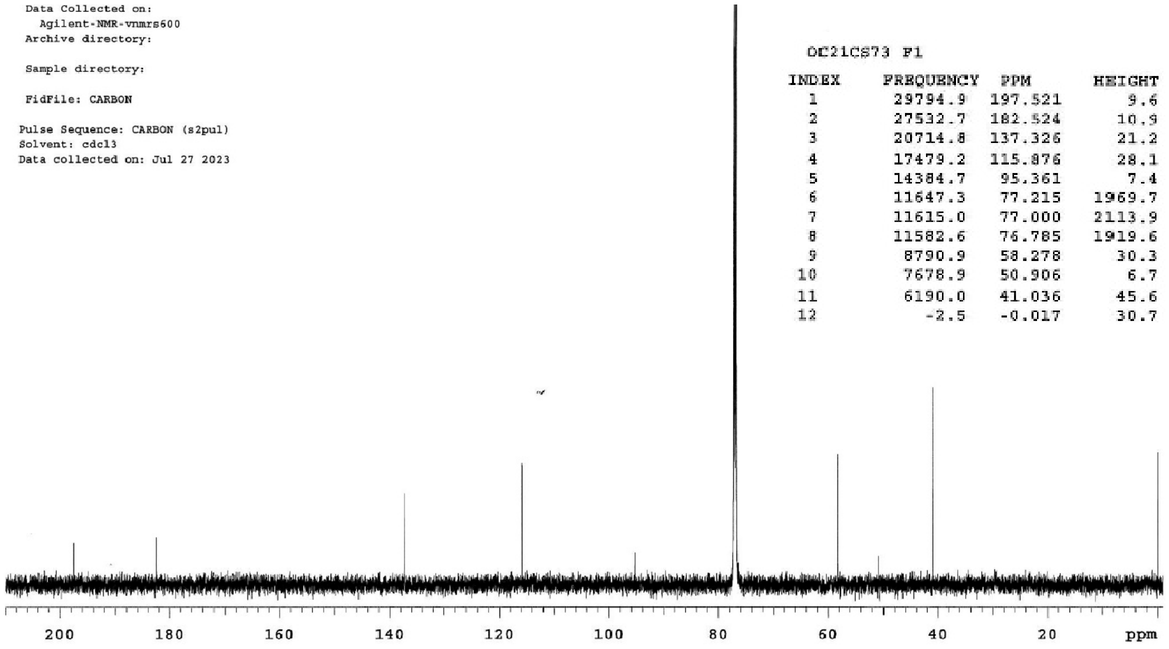

Figure SI-19: ^{1}H-NMR spectrum of compound **6** (600 MHz, DMSO-d_6)

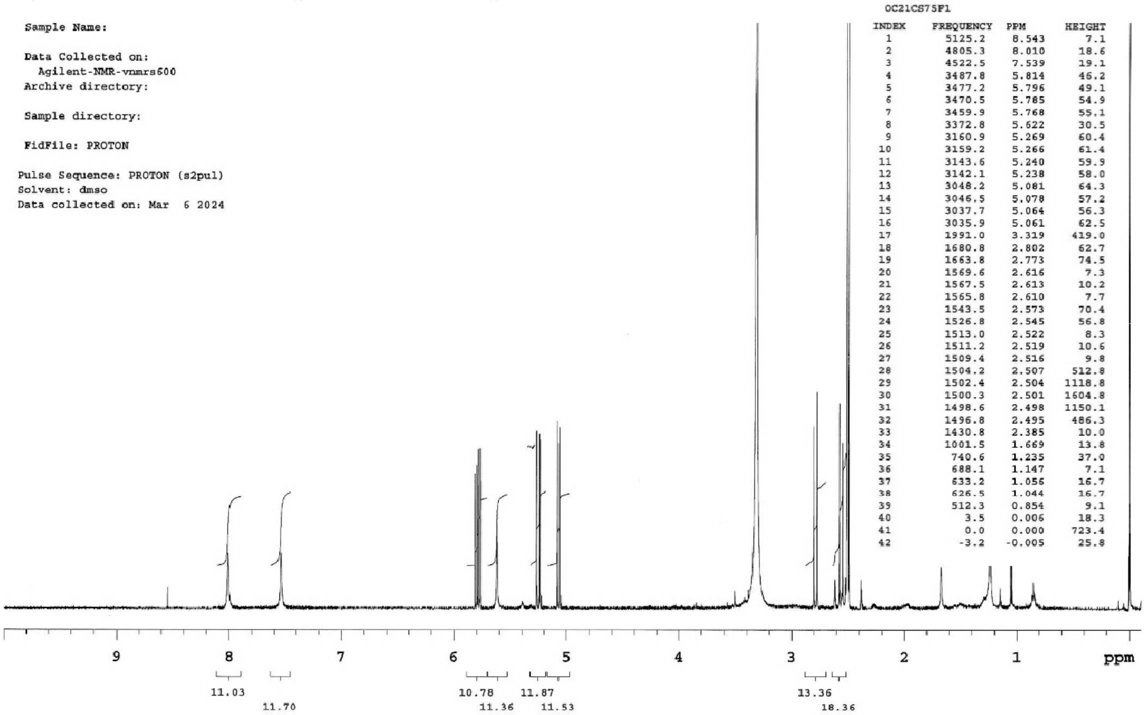

Figure SI-20: ^{13}C-NMR spectrum of compound **6** (150 MHz, DMSO-d_6)

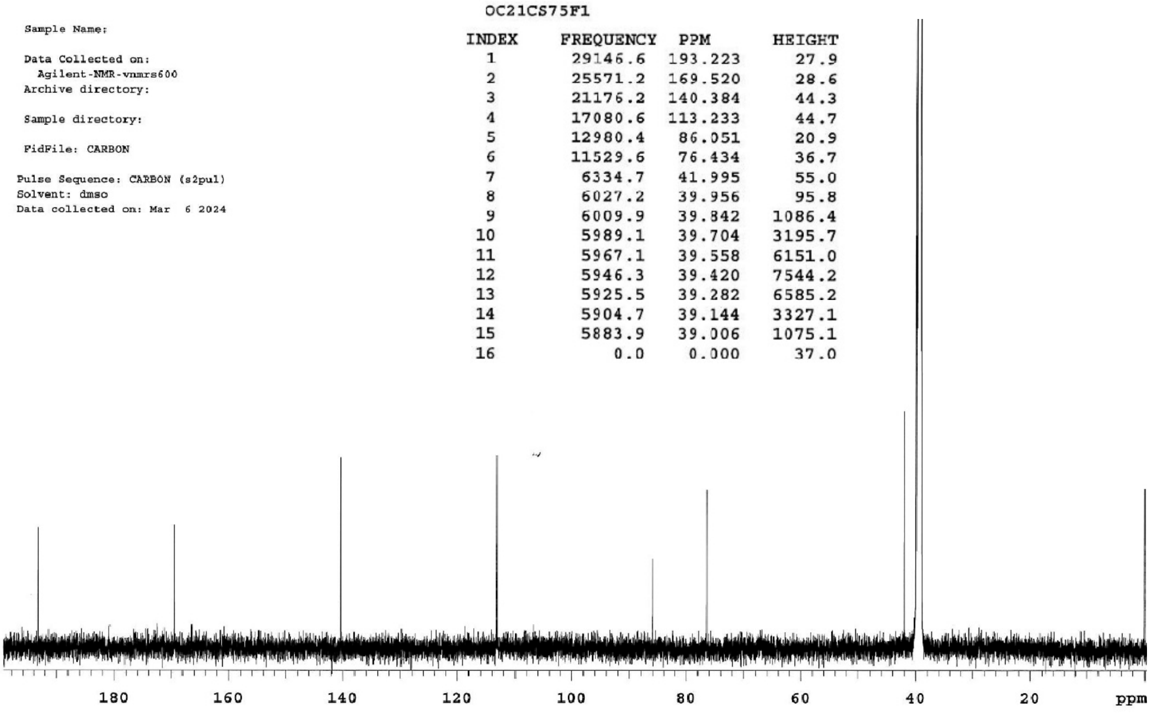

Figure SI-21: ^{1}H-NMR spectrum of compound **14** (600 MHz, $CDCl_3$)

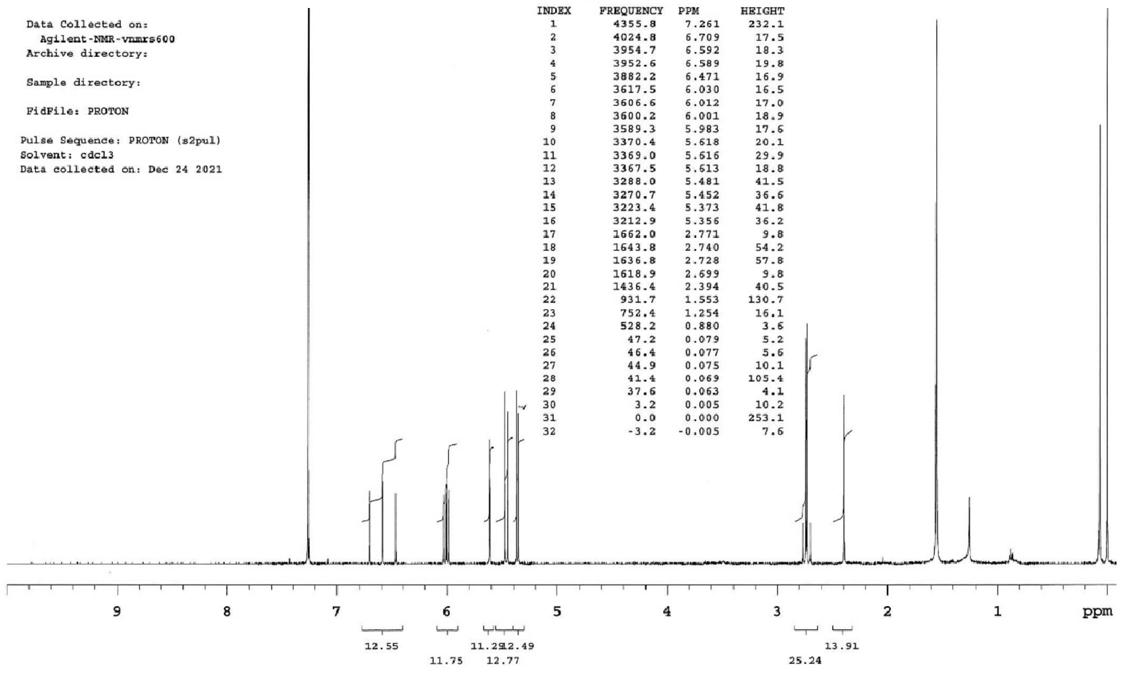

Figure SI-22: ^{13}C-NMR spectrum of compound **14** (150 MHz, $CDCl_3$)

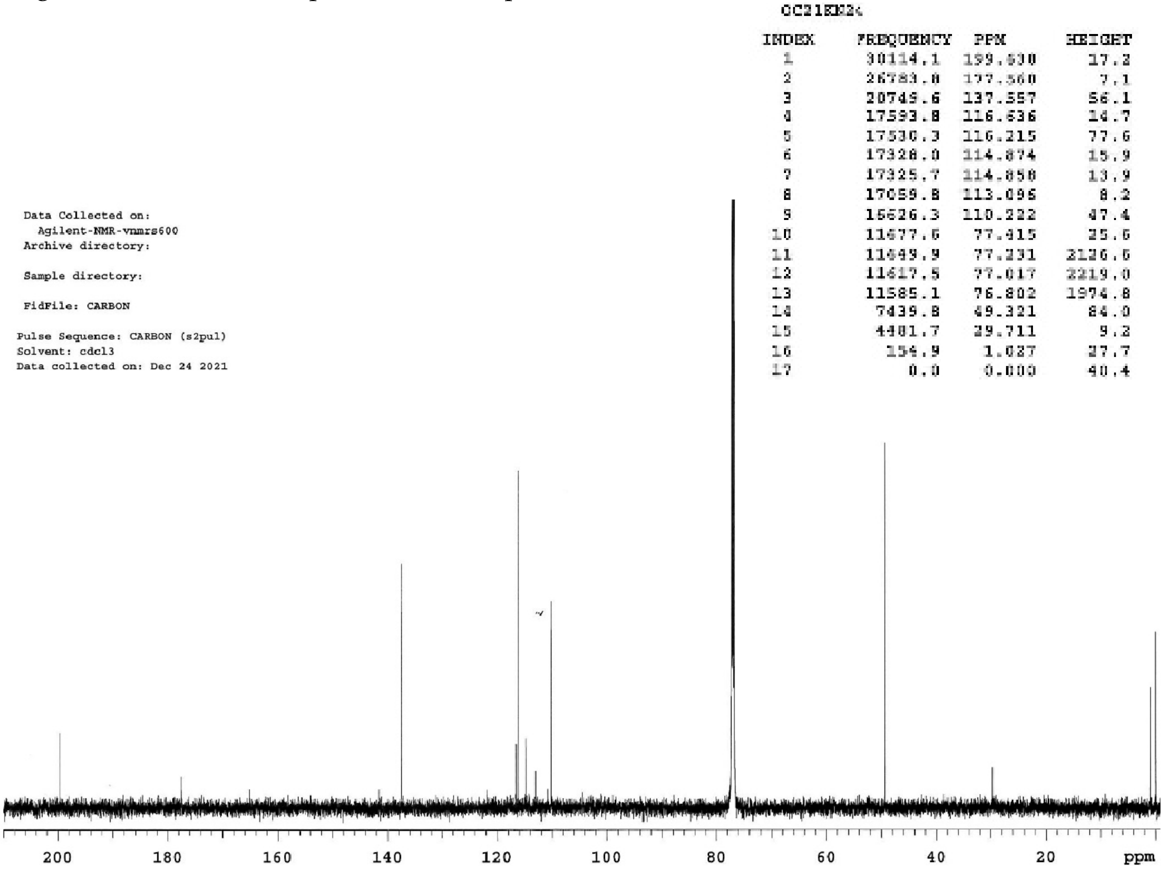

Supplement: Supplementary file 1 [file marinedrugs-23-00449-s001.zip › marinedrugs-3943062-supplementary.pdf]
